# Venovenous extracorporeal membrane oxygenation in adult respiratory failure: Scores for mortality prediction

**DOI:** 10.1097/MD.0000000000003989

**Published:** 2016-06-24

**Authors:** Chun-Hsien Hsin, Meng-Yu Wu, Chung-Chi Huang, Kuo-Chin Kao, Pyng-Jing Lin

**Affiliations:** a Department of Cardiovascular Surgery; b Department of Thoracic Medicine, Chang Gung Memorial Hospital and Chang Gung University, Taoyuan, Taiwan, R.O.C.

**Keywords:** acute respiratory failure, ECLS, ECMO, ECMOnet score, RESP score, SOFA score

## Abstract

Despite a potentially effective therapy for adult respiratory failure, a general agreement on venovenous extracorporeal membrane oxygenation (VV-ECMO) has not been reached among institutions due to its invasiveness and high resource usage. To establish consensus on the timing of intervention, large ECMO organizations have published the respiratory extracorporeal membrane oxygenation survival prediction (RESP) score and the ECMOnet score, which allow users to predict hospital mortality for candidates with their pre-ECMO presentations. This study was aimed to test the predictive powers of these published scores in a medium-sized cohort enrolling adults treated with VV-ECMO for acute respiratory failure, and develop an institutional prediction model under the framework of the 3 scores if a superior predictive power could be achieved. This retrospective study included 107 adults who received VV-ECMO for severe acute respiratory failure (a P_a_O_2_/FiO_2_ ratio <70 mm Hg) in a tertiary referral center from 2007 to 2015. Essential demographic and clinical data were collected to calculate the RESP score, the ECMOnet score, and the sequential organ failure assessment (SOFA) score before VV-ECMO. The predictive power of hospital mortality of each score was presented as the area under receiver-operating characteristic curve (AUROC). The multivariate logistic regression was used to develop an institutional prediction model. The surviving to discharge rate was 55% (n = 59). All of the 3 published scores had a real but poor predictive power of hospital mortality in this study. The AUROCs of RESP score, ECMOnet score, and SOFA score were 0.662 (*P* = 0.004), 0.616 (*P* = 0.04), and 0.667 (*P* = 0.003), respectively. An institutional prediction model was established from these score parameters and presented as follows: hospital mortality (*Y*) = −3.173 + 0.208 × (pre-ECMO SOFA score) + 0.148 × (pre-ECMO mechanical ventilation day) + 1.021 × (immunocompromised status). Compared with the 3 scores, the institutional model had a significantly higher AUROC (0.779; *P* < 0.001). The 3 published scores provide valuable information about the poor prognostic factors for adult respiratory ECMO. Among the score parameters, duration of mechanical ventilation, immunocompromised status, and severity of organ dysfunction may be the most important prognostic factors of VV-ECMO used for adult respiratory failure.

## 1 Introduction

Extracorporeal membrane oxygenation (ECMO) is an effective respiratory support to correct hypoxemia in adult patients with severe acute respiratory failure (ARF), which is not improved by advanced mechanical ventilation (MV).^[1,2]^ According to the database of Extracorporeal Life Support Organization (ELSO) registered from 2000 to 2012, the overall salvage rate of ECMO for ARF in adults is 57%.^[3]^ The venovenous (VV) mode is the preferred mode of ECMO in this setting, and yields a significantly higher survival rate when compared with venoarterial (VA) ECMO (VV vs VA: prevalence 82% vs 23%; survival rate 60% vs 40%; *P* < 0.001).^[3]^ However, ECMO has not gained full acceptance among medical institutions worldwide due to its invasiveness and high resource usage.^[4,5]^ The fact that VV-ECMO itself fails to bring survival benefits in the conventional ventilatory support versus extracorporeal membrane oxygenation (CESAR) trial increases the controversy of VV-ECMO for ARF in adult patients.^[6]^ Furthermore, a delayed administration of VV-ECMO (in general, MV >7 days before ECMO) is a known risk factor that may turn this salvage therapy into futile medical care.^[7–9]^ Therefore, to establish consensus on the timing of intervention, the ELSO and other European ECMO organizations have retrospectively analyzed their databases of adult respiratory failure and launched some outcome-predicting scores to enhance the decision-making process; 2 of them are the respiratory extracorporeal membrane oxygenation survival prediction (RESP) score^[3]^ and the ECMOnet score.^[10]^ Except the 2 published scores, the sequential organ failure assessment (SOFA) score^[11]^ is also a common tool used to predict mortalities in adult ECMO.^[12–14]^ Since the therapeutic experience and equipment may significantly differ among institutions, physicians are encouraged to validate the predictive power of these “packaged” scores with their own databases before using them to recruit candidates.^[15,16]^ The primary aim of this study was to validate the predictive powers on hospital mortality of the 3 published models (RESP, ECMOnet, SOFA) with our patient cohort of adult respiratory ECMO, and the secondary aim was to develop an institutional prediction model under the framework of these packaged scores to see if a superior predictive power could be achieved.

## 2 Materials and methods

### 2.1 Study population

From March 2007 to April 2015, a total of 123 adult patients received VV-ECMO for advanced respiratory support at Chang Gung Memorial Hospital. This university-affiliated hospital is a tertiary referral center with 3400 beds. To reduce the heterogeneities in disease severity and patient management, 16 patients were excluded due to a requested transfer to another hospital (n = 1), switching to VA or mixed configuration of ECMO (n = 5), and death on VV-ECMO in the first 24 hours (n = 10, hemorrhagic shock soon after device implantation in 4 patients and shock without obvious hemorrhagic focus in 6 patients). Therefore, only 107 among the 123 patients who received a single run of VV-ECMO and survived on VV-ECMO for >24 hours were enrolled in this retrospective study. This study was conducted in accordance with the amended Declaration of Helsinki.^[17]^ The ethics committee of the Chang Gung Medical Foundation approved the protocol (CGMF IRB no. 104–6277B) and waived the necessity of individual patient consent.

### 2.2 The Institutional criteria for adult venovenous ECMO

Our indication of VV-ECMO was a deteriorating hypoxia (a P_a_O_2_/FiO_2_ ratio <70 mm Hg) under advanced MV (FiO_2_ >0.8 and peak inspiratory pressure [PIP] >35 cm H_2_O). Nevertheless, VV-ECMO was contraindicated in candidates showing (1) uncontrolled hemorrhages, (2) obvious brain damages (intracranial hemorrhages, infarctions, or mass), and (3) significant hemodynamic instability before the intervention.

### 2.3 Data collection

We retrospectively collected the demographic and clinical variables required for calculating the RESP score, ECMOnet score, and SOFA score before the administration of VV-ECMO in each patient, from our institutional electronic medical record (EMR) system. These medical records were registered from March 2007 to April 2015. The following variables were collected: age; sex; etiologies of ARF (viral pneumonia, bacterial pneumonia, asthma, trauma and burn, aspiration pneumonia, other acute respiratory diagnoses); immunocompromised status (hematologic malignancy, solid tumor, solid organ transplantation, liver cirrhosis Child B or C, or autoimmune diseases requiring long-term steroid or other immunosuppressive therapy)^[3,13]^; nonpulmonary infection; bicarbonate infusion; arterial blood gas measures; MV settings (MV duration, PIP and positive end-expiratory pressure [PEEP]); and the outcomes (weaning off VV-ECMO and surviving to discharge). For practical purposes, we made some differences in the RESP score to its original version. First, we defined patients having an “aspiration pneumonitis” rather than a “bacterial pneumonia” if the diagnosis of “aspiration” was made, although they had an identified pathogen in their sputum cultures before ECMO. Second, we assigned the patients with fungal pneumonia to the category of bacterial pneumonia. Third, we excluded 3 items (nitric oxide inhalation, neuromuscular blockade agents, and cardiac arrest before ECMO) in the calculation of RESP score. As our inclusion criteria of VV-ECMO for ARF in adults, patients of VV-ECMO should be paralyzed with neuromuscular blockade agents before ECMO and have a relative stable hemodynamics. We excluded the item of nitric oxide inhalation because the information of nitric oxide inhalation was often missing in our EMR system before 2012. Fourth, we assigned a SOFA neurological assessment score to each patient according to his/her neurological status before sedation (the assumed Glasgow Coma Scale),^[18]^ since all of them were sedated before VV-ECMO.

### 2.4 Practice of VV-ECMO in adults with respiratory failure

**Figure 1:**
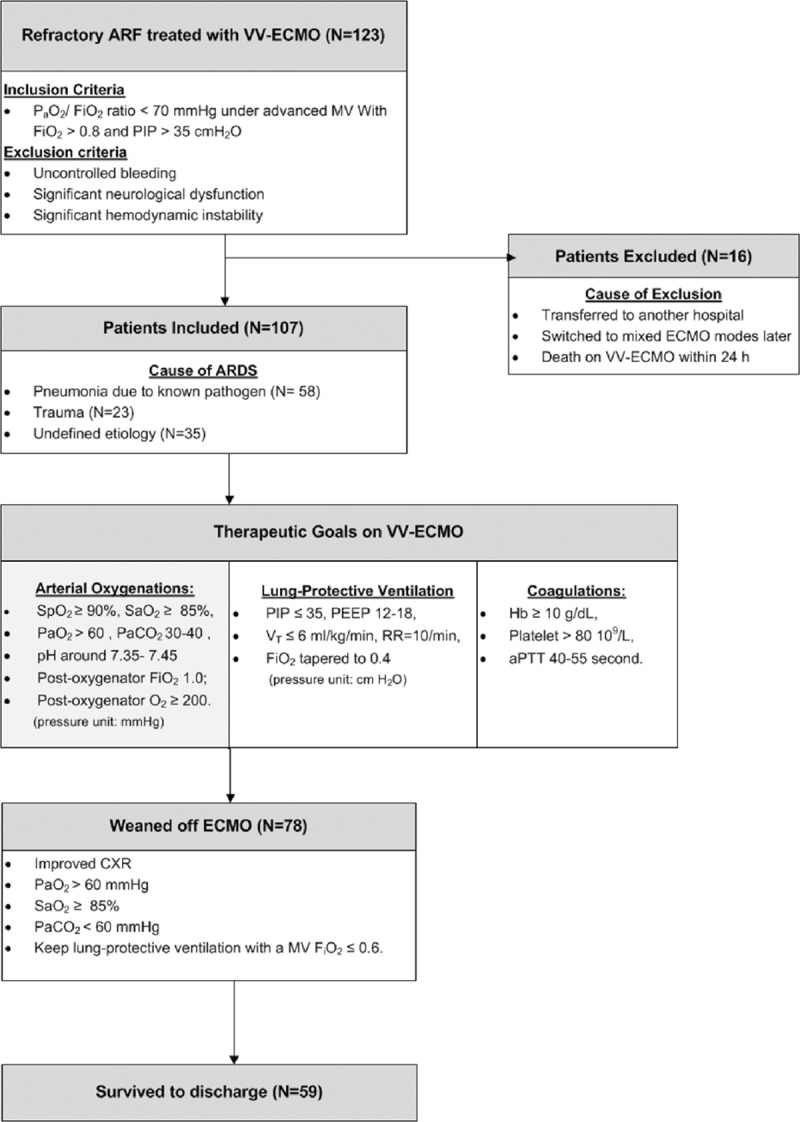
Flow chart of patient distribution and managements during venovenous extracorporeal membrane oxygenation. ARF = acute respiratory failure, FiO_2_ = fraction of inspired oxygen, PaO_2_ = arterial oxygen tension, PEEP = positive end-expiratory pressure, PIP = peak inspiratory pressure;, RR = respiratory rate, SaO_2_ = arterial oxygen saturation, SpO_2_ = oxyhemoglobin saturation by pulse oximetry, *V*_T_ = tidal volume, VV-ECMO = venovenous extracorporeal membrane oxygenation.

We have thoroughly described our techniques and therapeutic protocol of VV-ECMO in our previous publications.^[12,19,20]^Figure 1 summarizes the major therapeutic goals of our VV-ECMO. We use the Capiox emergent bypass system (Terumo Inc., Tokyo, Japan) and the 2 cannula method (DLP Medtronic, Minneapolis, MN; femoral inflow cannula: 19–23 French, jugular outflow cannula: 17–21 French) to perform VV-ECMO via percutaneous cannulation. Initially, we maximize the sweep gas flow (10 L/min, pure oxygen) to rapidly remove the CO_2,_ and gradually increase the ECMO pump flow to achieve a steady flow that carries the best pulse oximetry-detected oxyhemoglobin saturation (S_p_O_2_). To rest the lung on VV-ECMO, we change the setting of MV to a lung-protective level step by step. At first, we use a pressure-control mode with a PIP ≤35 cm H_2_O and a moderate PEEP (often 10–14 cm H_2_O) to obtain an estimated tidal volume ≤6 mL/kg/min on VV-ECMO. Then we take arterial and the postoxygenator blood samples to adjust the sweep gas flow and the MV F_i_O_2_. We also adjust the pump speed dynamically to provide a best S_p_O_2_ (>90%) and S_a_O_2_ (>85%) to gradually taper the PIP to 30 cm H_2_O and MV F_i_O_2_ to 0.4. The hemoglobin is kept at a level of ≥10 g/dL to increase the capacity of oxygenation. Keeping a modest anticoagulation on VV-ECMO with systemic heparinization is necessary except in the hemorrhagic patients. The therapeutic range of activated partial thromboplastin time is 40 to 55 seconds. In patients showing significant improvements, we would try to wean the patient from VV-ECMO as long as the arterial oxygenation could be maintained under the lung-protective ventilation, with a MV F_i_O_2_ ≤0.6.

### 2.5 Statistical analysis

Statistical analyses were performed with SPSS for Windows (Version 15.0, SPSS, Inc., IL). For all analyses, the statistical significance was set at *P* < 0.05. The independent *t* test was used for univariate comparisons of the independent numerical variables. If the numerical variables were not normally distributed, the Mann–Whitney *U* test was used for univariate comparisons. The chi-square or Fisher exact test was used to compare the categorical variables. Data were presented as mean ± standard deviation (SD) for numerical variables with normal distribution or median (interquartile range) for numerical variables without normal distribution. The categorical data were presented as number (percentage). The predictive power of hospital mortality of each score was presented as the area under receiver-operating characteristic curve (AUROC). The AUROCs among prediction models were compared with a nonparametric approach suggested by Hanley and McNeil.^[21]^ The multivariate logistic regression method with backward stepwise selection was used to develop the institutional mortality prediction model.^[22]^ All variables with a *P* < 0.05 in univariate tests were tested by the multivariate logistic regression analysis. Statistical significance was assessed at the level of *P* < 0.05. The Hosmer–Lemeshow test was used to assess goodness of fit for the institutional model. The Kaplan–Meier method was used to calculate the survival on VV-ECMO. The endpoint for the survival analysis was death on VV-ECMO.

## 3 Results

### 3.1 Univariate comparisons

**Figure 2:**
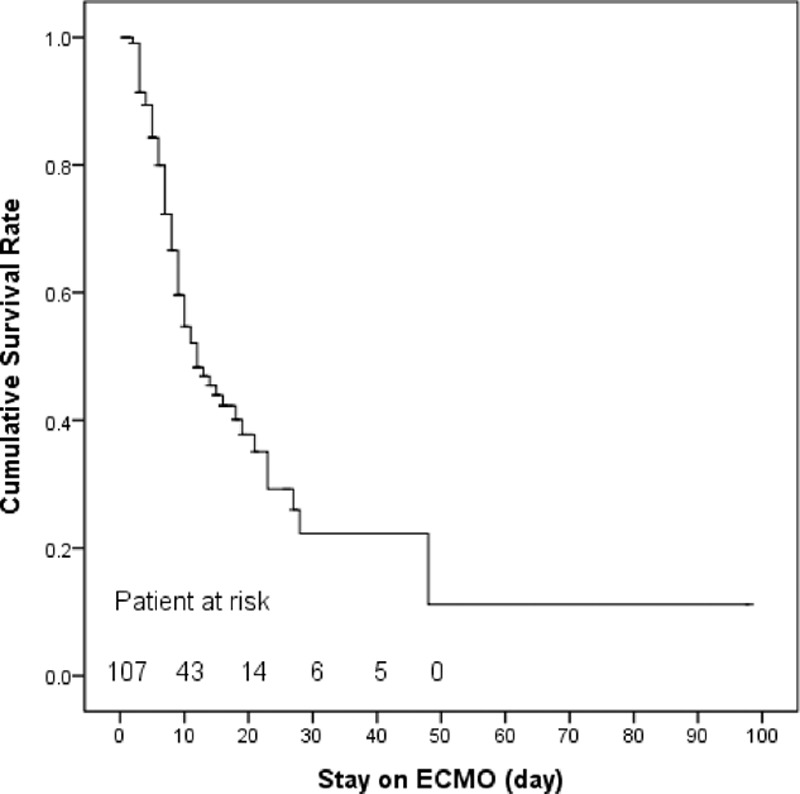
Survival curve on venovenous extracorporeal membrane oxygenation (ECMO).

The mean age of the 107 patients was 55 ± 16 years and 73% (n = 78) of them were male. Figure 1 shows the flowchart of patient distribution. The etiologies of ARF were categorized into 5 groups: bacterial pneumonia (n = 36; 3 were fungal pneumonia, and the top 3 bacteria were *Staphylococcus aureus*, *Pseudomonas aeruginosa*, and *Acinetobacter baumannii*); viral pneumonia (n = 18; all influenza A); trauma and inhalation injury (n = 21); aspiration pneumonitis (n = 3; 2 after surgeries of gastrointestinal tract and 1 had tracheoesophageal fistula); and others (n = 29; 16 were pneumonia without identifiable pathogens, 4 were pulmonary hemorrhage caused by autoimmune vasculitis, 7 were pulmonary edema in patients with chronic renal failure or after cardiac interventions, 1 was neurogenic pulmonary edema after cerebral aneurysm intervention, and 1 was pneumonitis after near-drowning). The median duration of MV before VV-ECMO was 3 (1–8) days. The mean values of pre-ECMO SOFA score, RESP score, and ECMOnet score were 11 ± 2, 0 ± 3, and 5 ± 2, respectively. Seventy-three per cent (n = 78) of the patients were weaned off VV-ECMO and 55% (n = 59) of them survived to hospital discharge. Eight patients died for the major hemorrhagic complications (intracranial hemorrhages in 3 patients, intra-abdominal/retroperitoneal hemorrhages in 2 patients, diffuse mucosal bleedings in 2 patients, and hemothorax in 1 patient) during the support of VV-ECMO. The other nonsurvivors (n = 40) showed a dependence on respiratory supports, either VV-ECMO or MV, and died with sepsis or multiple organ failure. The median values of ECMO stay and hospital stay were 9 (5–15) days and 43 (26–47) days, respectively. The actual survivals of RESP score were 75% in class I (score ≥6), 68% in class II (score 3–5), 63% in class III (score −1 to 2), 24% in class IV (score −5 to -2), and 38% in class V (score ≥−6) in this study. Figure 2 demonstrates the survival curve on VV-ECMO. Table 1 shows the results of univariate comparisons of the score parameters between the survivors and nonsurvivors. With ROC curve analysis, the discriminative powers on hospital mortality of the SOFA score, RESP score, and ECMOnet score were all significant but poor in our patients (AUROC: 0.667, 0.662, and 0.616, respectively).

**Table 1:**
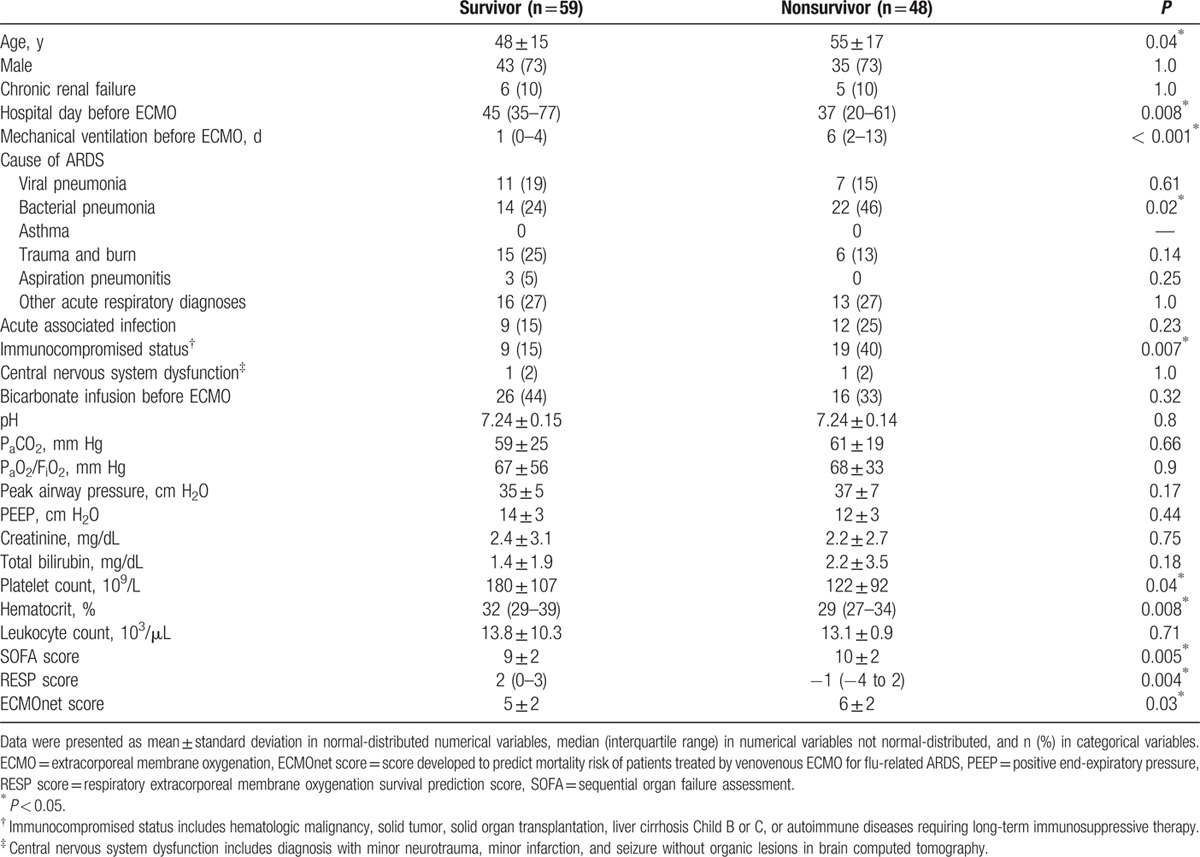
Information before venovenous extracorporeal membrane oxygenation.

### 3.2 Multivariate analysis

Since the published scores showed unsatisfactory results, an institutional mortality prediction model was developed with the multivariate logistic regression analysis. Table 2 shows the pre-ECMO variables associated with hospital mortality in multivariate analysis. The institutional mortality prediction model was presented as follows: Predicted mortality (*Y*) = е^*X*^/(1 + е^*X*^); *X* = −3.173 + 0.208 × (SOFA score before VV-ECMO) + 0.148 × (MV day before VV-ECMO) + 1.021 × (immunocompromised status). The model explained 33.3% (Nagelkerke *R*^2^) of the variance in hospital mortality and correctly classified 69.2 % of the cases (sensitivity: 78.0%; specificity: 58.3 %). This predictive model also well-fitted the dataset (Hosmer–Lemeshow test: *χ*^2^ = 10.4, *P* = 0.17). Table 3 shows the discriminative (predictive) powers of the 4 models (the current model, the SOFA score, the RESP score, and the ECMOnet score). After pair-wise comparisons, the AUROCs of the 3 scores were similar in statistics, and the current model had a significantly higher AUROC than the other 3 scores in this cohort. Table 4 shows the scales of mortality estimated by the current model.

**Table 2:**
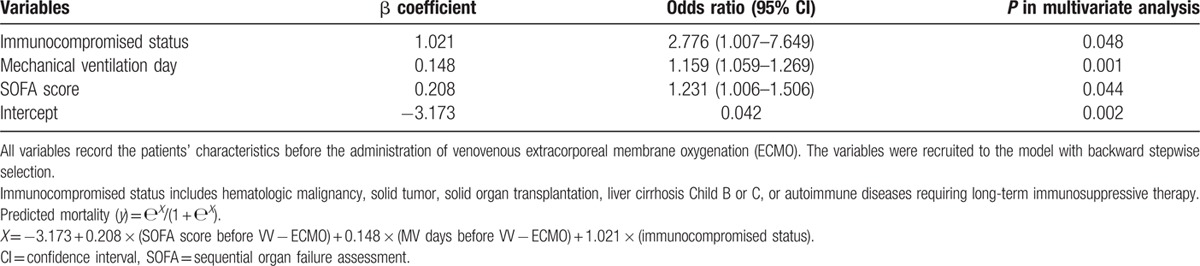
The variables associated with hospital mortality in multivariate analysis.

**Table 3:**
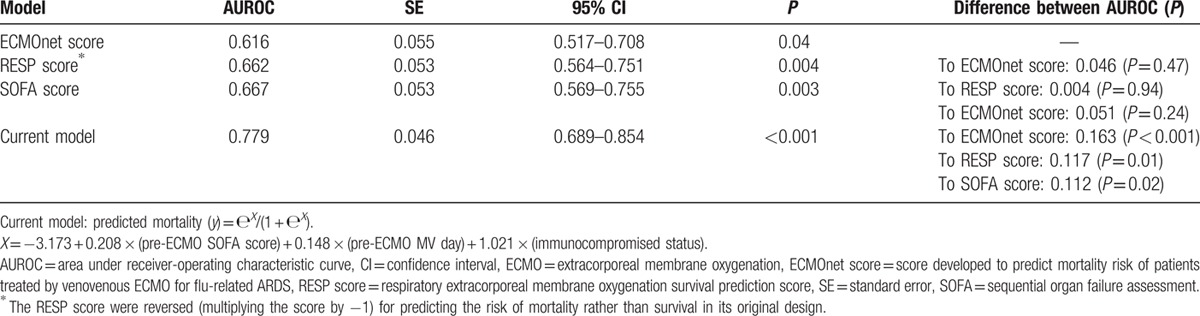
Discriminative power of scoring systems on hospital mortality.

**Table 4:**
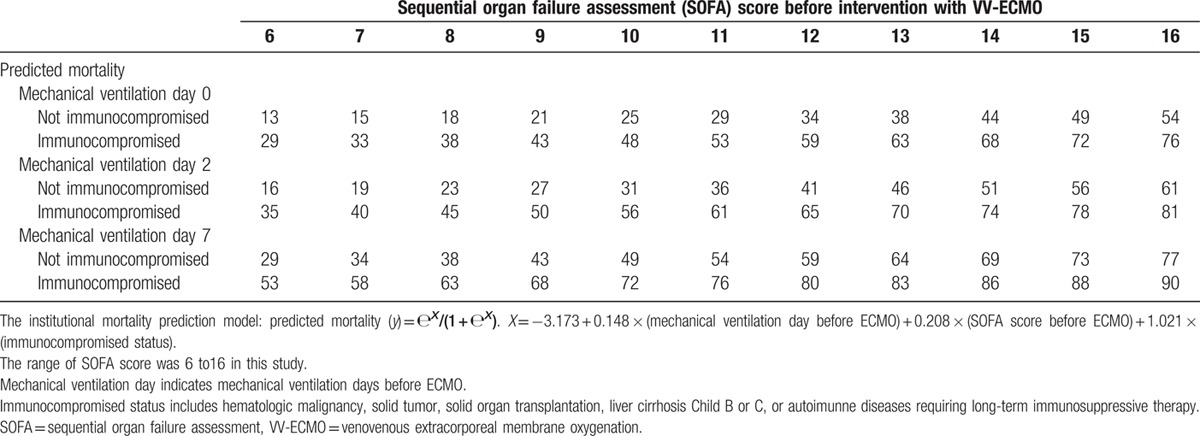
Scale of predicted hospital mortality rates (%) with venovenous extracorporeal membrane oxygenation in adult patients with acute respiratory failure.

## 4 Discussion

The study was aimed to find a practical model to predict hospital mortality in adult patients receiving VV-ECMO for severe ARF. Such a tool may theoretically improve the successful rate of this resource-demanding therapy by excluding the poor-prognostic candidates from VV-ECMO, or by actively recruiting the good-prognostic candidates before clinical deterioration. To meet this purpose, we started to search the ideal model among the published scores that have been used for mortality prediction in adult respiratory ECMO. We chose 3 scoring systems (RESP score, ECMOnet score, and SOFA score) that estimate hospital mortalities based on patients’ characteristics presented before ECMO. The RESP score and the ECMOnet score are specific designed for adult respiratory ECMO and launched recently.^[3,10]^ Both of them are derived from multicenter databases and verified with rigorous statistical methodologies. We also incorporated the SOFA score and compared its predictive power with the above mentioned scores, as the authors did in their original studies.^[3,10]^

However, all the 3 scores showed a real but unsatisfactory predictive power of hospital mortality in this medium-sized cohort. This was not surprising since similar results are shown in some recently published single-center studies of VV-ECMO^[13,14,23]^ (Table 5). In our opinions, the “expectation gap” between the actual and the expected performance of these scores may be mainly generated from 2 sources: the cohort composition and the score parameters. The differences of cohort composition among the single-center studies and the original studies producing the 2 scores should be the first source of this “expectation gap.” According to Table 5, a scoring system often performs better in its original cohort than in the validated cohort.^[16]^ Some equivocal results are shown in 2 studies performing external validation of RESP score and not well-explained by the authors.^[3,23]^ In fact, the tested cohorts could never be exactly the same with the original cohorts that produce these “packaged” scores, and some adjustments of the original scores may be necessary to fit the clinical demands among institutions. For example, the RESP score has a complex classification of the diagnoses in ARF and this may distort the score assigned to each etiology in a median-sized cohort. In its original cohort, more than half of the patients (1371 in 2355) belonged to undetailed categories of “others acute respiratory diagnoses” and “other (diagnosis).”^[3]^ All of the definite diagnoses (bacterial pneumonia, viral pneumonia, aspiration pneumonitis, asthma, and trauma) have similar survival rates, from 64% to 70%.^[3]^ The overall survival rate of patients with definite diagnoses is significantly higher than the overall survival rate of the patients with undetailed diagnoses (68% vs 49%; *P* < 0.001, recalculated).^[3]^ Thus, RESP score may become more attractive to physicians if it has a simplified classification for the diagnoses in ARF. Except the complex definitions in score parameters, these “packaged” scores may also have decreased predictive powers in a specific disease because of neglecting factors which have significant impacts on prognosis. For example, studies using SOFA score to predict hospital mortality in ECMO-treated patients often yield unsatisfactory results. An obvious disadvantage of using SOFA score to predict hospital mortality in adult respiratory ECMO is not taking the length of pre-ECMO MV into consideration, which is an important poor-prognostic factor in this patient group.^[9]^

**Table 5:**
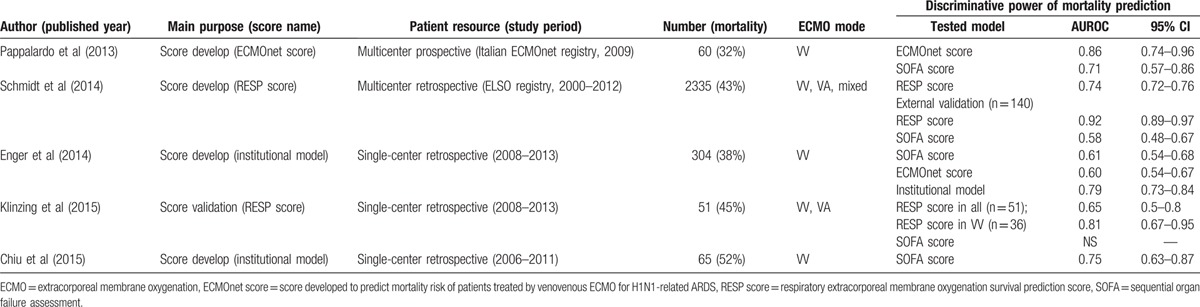
Recent publications focused on developing or validating a mortality prediction model in adult respiratory extracorporeal membrane oxygenation with receiver operating characteristic curve analysis.

Despite none of the 3 published scores performing perfectly in this study, they do help physicians to see the big picture of adult respiratory ECMO. Rather than their predictive powers of mortality in a specific cohort, these published scores are more important in providing the comprehensive knowledge of important prognostic factors for adult respiratory ECMO. These scores therefore may guide investigators to find their future directions for outcome improvement in a more efficient way. During the validation process of the 3 packaged scores, we also needed to perform a detailed analysis of all the parameters included in the scores. We found that the duration of MV, the immunocompromised status, and the SOFA score were independent predictors of hospital mortality in adult patients who were ready to be treated with VV-ECMO. Therefore, we built our institutional model with the 3 independent predictors and yielded an increased discriminative power than the original scores in this cohort. The model could be easily applied at bedside with a table demonstrating the estimated risks of mortality according to the combinations of the 3 predictors (Table 4). Based on our results, we agreed the viewpoint that VV-ECMO should not be recommended for treating ARF in immunocompromised patients with prolonged pre-ECMO MV and multiple organ failure,^[9]^ since the mortality rate may be extremely high.

The major limitations of this study are its retrospective design and limited sample size. It was a pilot study and the model would need to be validated in different settings and populations. It did not provide a comprehensive discussion of adult respiratory ECMO since some high-risk patients were excluded to increase the homogeneity in patient management. These high-risk groups were patients who died quickly on VV-ECMO and patients who required VA or mixed configurations of ECMO. Patients who died quickly on VV-ECMO often showed refractory hypotension soon after the device implantation, and there is often insufficient evidence to draw a solid conclusion about the cause of their deaths or to predict it. Patients requiring VA or mixed configurations of ECMO often have an advanced disease and showed unstable hemodynamics due to a combined cardiopulmonary failure.^[2,4]^ Many studies have separated these patients from the discussion of adult respiratory ECMO since they have a different risk of hospital mortality than the patients who could be stabilized by VV-ECMO.^[7,13,14]^ In fact, no matter what configuration of ECMO is chosen, the prime task of the ECMO specialists is to quickly stabilize the patient. A pre-ECMO echocardiography is very helpful in deciding the ECMO configuration.^[24,25]^ The VA rather than VV-ECMO should be used in patients showing a strained right ventricle with significant tricuspid regurgitation in bedside echocardiography. The VA-ECMO directly improves the arterial oxygenation and provides a better perfusion of vital organs than VV-ECMO. However, since the VV-ECMO provides a prepulmonary gas exchange and is more physiological, the VA-ECMO may need to be changed to VV configuration after a couple of days if the native lungs are not well recovered.^[26]^ Therefore, heart–lung interactions before and during different configurations of ECMO should be an important direction of research to define the optimal timing and configuration of adult respiratory ECMO in specific scenarios.^[27]^

## 5 Conclusions

The RESP score, the ECMOnet score, and the SOFA score provide valuable information about the poor-prognostic factors for adult respiratory ECMO. Nevertheless, some adjustments of the score parameters may be necessary to fit the clinical demand among institutions. Among the score parameters, duration of MV, immunocompromised status, and severity of organ dysfunction may be the most important prognostic factors of VV-ECMO used for adult respiratory failure.
